# Supplementary material for: Effects of High Voltage Electrical Discharge (HVED) on Endogenous Hormone and Polyphenol Profile in Wheat
Source: Plants (Basel). 2023 Mar 8;12(6):1235. doi: 10.3390/plants12061235 (PMC10054893; doi:10.3390/plants12061235)
Supplement: Supplementary file 1 [file plants-12-01235-s001.zip › Supplementary Tables S1-S9.pdf]

Supplementary Table S1. Analysis variance of germination (from 1. to 5. days), shoot and root length (from 2. till 5. days) under control and high voltage electrical discharge (HVED) treatment. Mean squares followed by asterisks (*) are significantly different (P<0.05). Analysis included five repetitions for germination, shoot, and root length.

Source of variation	Df	Shoot	Root	Source of variation	Df	Germination
Treatment (T)	1	17.3*	39.4*	Treatment (T)	1	1109.4*
Day (D)	3	855.4*	1089.3*	Day (D)	2	700.5*
(T*D)	3	2.7*	1.5*	(T*D)	2	673.4*
Error: 396	19	0	1	Error: 54	52	1

* significant at P≤0.05

Supplementary Table S2. Analysis variance of hormone accumulation in shoot under control and high voltage electrical discharge (HVED) treatment for 2nd and 5th day. Mean squares followed by asterisks (*) are significantly different (P<0.05). Analysis included three-five repetitions.

Source of variation	Df	ABA	PA	*d*HPA	JA	JA-Le_Ile	IAA	GA4	GA20	GA3	GA1	GA8
Treatment (T)	1	0.0ns	0.0ns	0.0ns	24921.8*	1114.3*	0.0ns	0.0ns	1.4ns	0.0ns	0.3ns	0.0ns
Day (D)	1	2.5*	0.6*	0.2*	77958.4*	1076.8*	114.1*	6.1*	180.5*	4.3*	2.1*	185.1*
(T*D)	1	0.0ns	0.0ns	0.0ns	520.2ns	0.8ns	0.1ns	0.0ns	1.0ns	0.0ns	0.1ns	0.2ns
Error	9,13,14	0.1	0.0	0.0	1398.7	85.3	0.4	0.0	0.4	0.0	0.1	0.1

* significant at P≤0.05; ns-not significant

9(GA1, JA); 13(ABA, *d*HPA, GA20); 14(PA, GA4, GA3; GA8, JA-Le-Ile, IAA)

Supplementary Table S3. Analysis variance of polyphenol accumulation in shoot under control and high voltage electrical discharge (HVED) treatment for 2nd and 5th day. Mean squares followed by asterisks (*) are significantly different (P<0.05). Analysis included three-five repetitions.

Source of variation	Df	CA	*p*-CA	CFA	FA	*p*HBA	3,4-*d*HBA	2,6-*d*HBA	SA	BA
Treatment (T)	1	20.6*	1518.4*	119.3*	881843.7ns	164.7ns	18.8ns	0.6ns	117.2ns	11589.4*
Day (D)	1	1216.9*	397.1ns	660.7*	104485972.6*	68040.0*	3758.4*	6652.6*	10700.4*	1037071.4*
(T*D)	1	42.5*	268.2ns	3.2ns	631379.2ns	2.7ns	7.8ns	18.1ns	62.0ns	44251.5*
Error	11,12, 13,14	2.6	141.1	17.9	263890.6	512.4	14.2	7.1	70.4	772.7

* significant at P≤0.05; ns-not significant

11(FA, BA); 12(2,6-*d*HBA, SA); 13(CA, *p*-CA.); 14(*p*HBA, 3,4-*d*HBA, CFA)

Supplementary Table S4. Analysis variance of hormone accumulation in root under control and high voltage electrical discharge (HVED) treatment for 2nd and 5th day. Mean squares followed by asterisks (*) are significantly different ($P<0.05$). Analysis included three-five repetitions.

Source of variation	Df	ABA	PA	*d*HPA	GA1	GA8	JA-Le-Ile	IAA	JA
Treatment (T)	1	1.8*	0.1*	0.1*	1.0*	0.0ns	239.0ns	3.7ns	100.2ns
Day (D)	1	69.9*	1.9*	10.0*	2.5*	5.0*	35356.9*	1.1ns	1095869.4*
(T*D)	1	2.8*	0.1*	0.0ns	2.6*	0.0ns	776.5ns	1.3ns	3615.4ns
Error	9,11,14	0.2	0.0	0.0	0.0	0.0	755.5	2.7	4175.9

* significant at P≤0.05; ns-not significant

9(GA1); 11(ABA, *d*HPA, JA); 14(PA, GA8, JA-Le-Ile, IAA)

Supplementary Table S5. Analysis variance of polyphenol accumulation in root under control and high voltage electrical discharge (HVED) treatment for 2nd and 5th day. Mean squares followed by asterisks (*) are significantly different (P<0.05). Analysis included three-five repetitions.

Source of variation	Df	CA	*p*-CA	CFA	FA	*p*HBA	3,4-*d*HBA	2,6-*d*HBA	SA	BA
Treatment (T)	1	147.0ns	5170.3ns	0.0ns	31269.4*	2079.4*	21.2ns	45.4ns	66.8ns	213490.0*
Day (D)	1	45811.2*	7.1ns	0.9*	425014.9*	370.5ns	79.4*	60.6*	188.8*	194969.4*
(T*D)	1	69.5ns	4.8ns	0.1ns	20064.9*	606.3ns	167.5*	5.9ns	74.7ns	104573.9*
Error	10,11,13,14	116.9	1167.4	0.1	1730.1	177.2	15.5	11.1	19.8	3405.7

* significant at P≤0.05; ns-not significant

10(FA); 11(BA, CA, *p*-CA); 13(3,4-*d*HBA, SA); 14(CFA, *p*HBA, 2,6-*d*HBA)

Supplementary Table S6. Amount of hormones in shoot and root under control (0) and high electrical voltage discharge (HVED) treatment (H) on the 2nd and 5th day. Values represent means ± standard deviation. Analysis included three-five repetitions.

Treatment	Hormone	Shoot (2nd day)	Shoot (5th day)	Root (2nd day)	Root (5th day)
0	ABA	4.32 ± 0.4	3.48 ± 0.2	6.52 ± 0.4	2.98 ± 0.3
0	PA	0.88 ± 0.1	0.44 ± 0.1	0.51 ± 0.1	0
0	*d*HPA	0.26 ± 0.0	0	1.87 ± 0.1	0.27 ± 0.0
0	GA4	1.14 ± 0.2	0	0	0
0	GA20	8.11 ± 0.4	0.65 ± 0.2	0	0
0	GA3	1.53 ± 0.2	0.62 ± 0.3	0	0
0	GA1	10.06 ± 0.2	9.11 ± 0.2	7.53 ± 0.3	5.75 ± 0.1
0	GA8	7.46 ± 0.3	1.12 ± 0.4	1.05 ± 0.2	0
0	JA	321.5 ± 9.3	152.67 ± 30.4	759.33 ± 89.4	183.25 ± 29.0
0	JA_le_Ile	24.36 ± 4.7	8.94 ± 5.4	121.55 ± 23.3	19.14 ± 7.0
0	IAA	13 ± 0.8	7.66 ± 0.3	19.38 ± 2.5	18.34 ± 1.5
H	ABA	4.22 ± 0.2	3.49 ± 0.1	8.11 ± 0.6	2.82 ± 0.4
H	PA	0.86 ± 0.1	0.53 ± 0.0	0.80 ± 0.1	0
H	*d*HPA	0.21 ± 0.0	0	1.74 ± 0.1	0
H	GA4	1.25 ±1.2	0	0	0
H	GA20	6.42 ± 0.8	0.57 ± 0.1	0	0
H	GA3	1.52 ± 0.2	0.42 ± 0.1	0	0
H	GA1	10.22 ± 0.4	9.56 ± 0.3	6.06 ± 0.3	6.08 ± 0.1
H	GA8	7.67 ± 0.4	0.86 ± 0.1	1.06 ± 0.1	0
H	JA	397 ± 7.9	253.67 ± 25.5	733.25 ± 74.6	219.75 ± 30.1
H	JA_le_Ile	40.92 ± 14.6	24.63 ± 8.2	101 ± 46.5	25.03 ± 6.1
H	IAA	12.74 ± 0.7	7.77 ± 0.1	19.74 ± 1.3	19.8 ± 1.2

Abbreviations: Abscisic acid (ABA); phaseic acid (PA); dihydrophaseic acid (*d*HPA); gibberellic acid (GAs) derivates; jasmonic acid (JA); jasmonoyl-leucine-isoleucine (JA_Le_Ile); auxin (IAA).

Supplementary Table S7. Amount of polyphenols in shoot and root under control (0) and high electrical voltage discharge (HVED) treatment (H) on the 2nd and 5th day. Values represent means ± standard deviation. Analysis included three-five repetitions.

Treatment	Polyphenols	Shoot (2nd day)	Shoot (5th day)	Root (2nd day)	Root (5th day)
0	CA	17.14 ± 0.7	3.08 ± 0.9	135.33 ± 23.7	17.84 ± 3.9
0	*p*-CA	103.56 ± 14.1	101.8 ± 11.2	121.2 ± 48.0	108.5 ± 17.5
0	CFA	31.24 ± 1.5	44.52 ± 2.6	1.92 ± 0.5	1.64 ± 0.3
0	FA	863 ± 17.3	5786.67 ± 398.0	562.33 ± 8.9	286.75± 57.7
0	*p*HBA	296.8 ± 19.0	422 ± 23.3	78.35 ± 9.5	80.9 ± 14.8
0	3,4-*d*HBA	44.34 ± 4.9	16.08 ± 3.3	25.43 ± 3.4	23.46 ± 5.6
0	2,6-*d*HBA	1.82 ± 0.7	45.44 ± 3.5	7.02 ± 0.9	4.48 ± 0.9
0	SA	7.12 ± 1.8	63.7 ± 9.9	14.73 ± 0.8	17.26 ± 3.9
0	BA	581.5 ± 20.7	161 ± 36.1	255.33 ± 39.7	296 ± 72.4
H	CA	22.62 ± 2.6	2.09 ± 0.7	137.33 ± 4.6	28.65 ± 4.4
H	*p*-CA	131 ± 12.7	113 ± 5.0	158 ± 48.7	157.75 ± 12.9
H	CFA	37.38 ± 3.8	48.93 ± 8.8	2.12 ± 0.2	1.49 ± 0.4
H	FA	939.33 ± 4.9	6760 ± 586.9	734.33 ± 47.8	305.75 ± 29.4
H	*p*HBA	302.2 ± 26.9	429 ± 18.1	111.66 ± 11.1	90.85 ± 7.3
H	3,4-*d*HBA	43.6 ± 3.8	12.63 ± 0.6	16.87 ± 0.6	27.53 ± 3.7
H	2,6-*d*HBA	3.57 ± 1.2	42.87 ± 3.9	11.36 ± 6.0	6.52 ± 1.4
H	SA	16.62 ± 1.7	65.2 ± 14.7	14.5 ± 3.3	25.6 ± 2.8
H	BA	747 ± 32.3	107.55 ± 9.5	306.67 ± 50.9	704.75 ± 52.3

Abbreviations: Cinnamic acid (CA); *p*-coumaric acid (*p*-CA); ferulic acid (FA); caffeic acid (CFA); *p*-hydroxybenzoic acid (*p*HBA); 3,4-dihydroxybenzoic acid (3,4-*d*HBA); 2,6-dyhydroxybenzoic acid (2,6-*d*HBA); salicylic acid (SA) and benzoic acid (BA).

Supplementary Table S8. Factor loadings of shoot hormones and polyphenols under control and high voltage electrical discharge (HVED) treatment..

Variable	PC1	PC2	PC3
CA	0.052	0.003	0.066
IAA	0.054	0.001	0.004
BA	0.052	0.002	0.083
*p*HBA	0.053	0.003	0.000
SA	0.052	0.008	0.014
3,4-*d*HBA	0.053	0.004	0.004
2,6-*d*HBA	0.054	0.000	0.008
p-CA	0.012	0.229	0.211
CFA	0.044	0.062	0.005
FA	0.040	0.045	0.280
JA	0.035	0.103	0.108
ABA	0.053	0.004	0.012
PA	0.053	0.001	0.054
*d*HPA	0.052	0.006	0.020
JA-LE-ILE	0.029	0.153	0.001
GA 4	0.054	0.000	0.003
GA 20	0.052	0.008	0.017
GA 3	0.052	0.007	0.022
GA 1	0.047	0.030	0.085
GA 8	0.054	0.001	0.002
Germination (2nd day)	0.000	0.330	0.000
Shoot lenght	0.054	0.002	0.000
Explained variance (eigenvalue)	18.54	3.03	0.43
Proportion of total variance (%)	84.29	13.76	1.95
Cumulative variance (%)	84.29	98.05	100.00

PC1-3 (principal components)

*CA (cinnamic a.); *p*-CA (*p*-coumaric a.); CFA (caffeic a.); FA (ferulic a.); PA (phaseic a.); ABA (abscisic acid); PA (phaseic acid); *d*HPA (dihydrophaseic acid); GA (gibberelins 1,3,4,8,20); JA (jasmonic acid); JA-Le_Ile (jasmonoyl-leucine-isoleucine); IAA (auxin); *p*HBA (*p*-hydroxybenzoic acid); 3,4-*d*HBA (3,4-dihydroxybenzoic acid); 2,6-*d*HBA (2,6-dihydroxybenzoic acid); SA (salicylic acid); BA (benzoic acid)

Supplementary Table S9. Factor loadings of root hormones and polyphenols under control and high voltage electrical discharge (HVED) treatment.

Variable	PC1	PC2	PC3
CA	1.00	-0.01	0.01
IAA	1.00	0.00	0.00
BA	0.91	0.15	0.38
*p*HBA	-1.00	0.09	0.02
SA	-1.00	0.03	-0.02
3,4-*d*HBA	0.71	-0.70	-0.09
2,6-*d*HBA	-0.99	0.07	0.09
p-CA	0.78	0.61	0.14
CFA	-1.00	0.06	-0.06
FA	-0.99	0.10	-0.09
JA	0.98	0.04	-0.18
ABA	0.96	0.23	0.14
PA	0.65	0.74	0.16
*d*HPA	1.00	-0.07	-0.03
JA-LE-ILE	0.99	-0.01	-0.12
GA 20	-1.00	-0.03	0.09
GA 3	-0.97	-0.11	0.23
GA 1	0.66	-0.47	-0.59
GA 8	0.34	-0.44	0.83
Germination (2nd day)	-0.03	0.97	-0.22
Root lenght	-1.00	0.07	-0.04
Explained variance (eigenvalue)	16.66	2.91	1.43
Proportion of total variance (%)	79.34	13.85	6.81
Cumulative variance (%)	79.34	93.19	100.00

PC1-3 (principal components)

*CA (cinnamic a.); *p*-CA (*p*-coumaric a.); CFA (caffeic a.); FA (ferulic a.); PA (phaseic a.); ABA (abscisic acid); PA (phaseic acid); *d*HPA (dihydrophaseic acid); GA (gibberelins 1,3,4,8,20); JA (jasmonic acid); JA-Le_Ile (jasmonoyl-leucine-isoleucine); IAA (auxin); *p*HBA (*p*-hydroxybenzoic acid); 3,4-*d*HBA (3,4-dihydroxybenzoic acid); 2,6-*d*HBA (2,6-dihydroxybenzoic acid); SA (salicylic acid); BA (benzoic acid)
